# Effects of ingesting a pre-workout dietary supplement with and without synephrine for 8 weeks on training adaptations in resistance-trained males

**DOI:** 10.1186/s12970-016-0158-3

**Published:** 2017-01-03

**Authors:** Y. Peter Jung, Conrad P. Earnest, Majid Koozehchian, Minye Cho, Nick Barringer, Dillon Walker, Christopher Rasmussen, Mike Greenwood, Peter S. Murano, Richard B. Kreider

**Affiliations:** 1 Exercise & Sport Nutrition Lab, Department of Health & Kinesiology, Texas A&M University, College Station, TX 77843-4243 USA; 2 Nutrabolt, Bryan, TX 77807 USA; 3 Department of Health & Kinesiology, Center for Translational Research in Aging and Longevity, Texas A&M University, College Station, TX 77843-4243 USA; 4 Department of Nutrition and Food Sciences, Institute for Obesity Research & Program Evaluation, Texas A&M University, College Station, TX 77843 USA

**Keywords:** Ergogenic aids, Dietary supplement, Multi-ingredient supplement, Safety, Exercise performance, Cognitive function, Body composition

## Abstract

**Background:**

The purpose of this study was to examine whether ingesting a pre-workout dietary supplement (PWS) with and without synephrine (S) during training affects training responses in resistance-trained males.

**Methods:**

Resistance-trained males (*N* = 80) were randomly assigned to supplement their diet in a double-blind manner with either a flavored placebo (PLA); a PWS containing beta-alanine (3 g), creatine nitrate as a salt (2 g), arginine alpha-ketoglutarate (2 g), N-Acetyl-L-Tyrosine (300 mg), caffeine (284 mg), *Mucuna pruiriens* extract standardized for 15% L-Dopa (15 mg), Vitamin C as Ascorbic Acid (500 mg), niacin (60 mg), folate as folic acid (50 mg), and Vitamin B12 as Methylcobalamin (70 mg); or, the PWS supplement with *Citrus aurantium* extract containing 20 mg of synephrine (PWS + S) once per day for 8-weeks during training. Participants donated a fasting blood sample and had body composition (DXA), resting heart rate and blood pressure, cognitive function (Stroop Test), readiness to perform, bench and leg press 1 RM, and Wingate anaerobic capacity assessments determined a 0, 4, and 8-weeks of standardized training. Data were analyzed by MANOVA with repeated measures. Performance and cognitive function data were analyzed using baseline values as covariates as well as mean changes from baseline with 95% confidence intervals (CI). Blood chemistry data were also analyzed using Chi-square analysis.

**Results:**

Although significant time effects were seen, no statistically significant overall MANOVA Wilks’ Lambda interactions were observed among groups for body composition, resting heart and blood pressure, readiness to perform questions, 1RM strength, anaerobic sprint capacity, or blood chemistry panels. MANOVA univariate analysis and analysis of changes from baseline with 95% CI revealed some evidence that cognitive function and 1RM strength were increased to a greater degree in the PWS and/or PWS + S groups after 4- and/or 8-weeks compared to PLA responses. However, there was no evidence that PWS + S promoted greater overall training adaptations compared to the PWS group. Dietary supplementation of PWS and PWS + S did not increase the incidence of reported side effects or significantly affect the number of blood values above clinical norms compared to PLA.

**Conclusion:**

Results provide some evidence that 4-weeks of PWS and/or PWS + S supplementation can improve some indices of cognitive function and exercise performance during resistance-training without significant side effects in apparently health males. However, these effects were similar to PLA responses after 8-weeks of supplementation and inclusion of synephrine did not promote additive benefits.

**Trial registration:**

This trial (NCT02999581) was retrospectively registered on December 16th 2016.

## Background

Research has shown that ingestion of some nutrients and/or caffeinated beverages prior to exercise can improve mental focus and/or exercise capacity [1]. For this reason, a number of energy drinks and pre-workout supplements (PWS) have been developed and marketed to athletes. The primary ergogenic properties in most of these supplements appears to be water, carbohydrate, and caffeine [1]. However, more recently PWS’s have been developed that not only contain nutrients that may affect acute exercise performance (e.g., carbohydrate, caffeine, nitrates, etc.), but also nutrients that can increase energy expenditure, reduce catabolism, and promote protein synthesis thereby enhancing training adaptations when taken regularly during training (e.g., amino acids, creatine, β-alanine, etc.) [1–3]. Consequently, there has been increased interest in examining the acute and chronic safety and efficacy of PWS’s marketed to active individuals [4] as well as whether adding potentially ergogenic nutrients may promote additive benefits [1].

This study examined the safety and efficacy of daily ingestion of a market leading PWS on ratings of perception of readiness to perform, cognitive function, resting energy expenditure and metabolism, exercise performance, and markers of safety. The PWS studied contained several nutrients reported to have ergogenic properties including caffeine [5], beta-alanine [6], creatine [7], nitrate [8–11], arginine alpha-ketoglutarate [12] as well as other nutrients purported to affect cognitive function like tyrosine [13, 14] and Mucuna pruriens containing L-Dopa [15, 16]. It is well established that consuming caffeine prior to exercise (e.g., 3–6 mg/kg) can improve exercise performance, cognitive function, and vigilance [5]. A number of studies also indicate that ingestion of nitrate prior to exercise (e.g., 300 mg) can improve exercise capacity [9, 11, 17–20]. Theoretically, ingesting these nutrients at effective doses prior to exercise may improve cognitive function and vigilance leading to better workout performance. If so, regular use of these types of PWS’s may affect quality of training and/or training adaptations particularly if they contain nutrients that have been reported to enhance training adaptations like beta-alanine [6, 21–27] and/or creatine [7, 28].

*Citrus aurantium* is found in the peel of bitter orange and contains p-synephrine. *Citrus aurantium* (generally containing 20–100 mg of synephrine) has been purported to suppress appetite [29], increase resting energy expenditure and/or carbohydrate and fat oxidation rates [30–33] and promote weight loss [34–36] with no negative effects on the cardiovascular system [37–39]. There is also evidence that *Citrus aurantium* ingestion can affect memory [40, 41] and resistance-exercise performance [31]. Theoretically, adding *Citrus aurantium* to a PWS may promote greater resting energy expenditure, cognitive function, and/or exercise capacity during an exercise bout.

In an initial companion study that has been submitted separately upon editor request [42–44], we reported that acute ingestion of this PWS and PWS + S promoted greater changes in resting energy expenditure, perceptions of vigor and energy, and cognitive function scores compared to PLA. Therefore, the purpose of this study was to examine the effects of ingesting a market leading PWS with and without synephrine during 8-weeks of resistance-training on ratings of perception of readiness to perform, cognitive function, resting energy expenditure and metabolism, exercise performance, and markers of safety.

## Methods

This study was conducted as a prospective, randomized, double-blind, and placebo controlled cohort study. The study was conducted at the Exercise & Sport Nutrition Laboratory (ESNL) at Texas A&M University after obtaining approval from the university’s Human Participant Internal Review Board.

### Participant recruitment and familiarization

Apparently healthy, resistance-trained males were recruited to participate from local advertisements. Inclusion criteria required that each participant have at least 6 months of resistance training immediately prior to entering the study inclusive of performing bench press and leg press or squat. Participants were excluded if they presented with a history of treatment for metabolic disease, hypertension, thyroid disease, arrhythmias, and/or cardiovascular disease; and/or were currently using any prescription medication. Further exclusion criteria included an intolerance to caffeine and/or other natural stimulants; a history of smoking; and, excessive alcohol consumption (>12 drinks/week).

A total of 213 individuals responded to advertisements to participate in this study. Participants who met initial study entry criteria via phone interview or online questionnaire screening were invited to a familiarization session where the details of the study were explained, informed consent was obtained, medical history was assessed, a fasting blood sample was obtained, and a general medical exam was performed by a registered nurse to determine eligibility to participate in the study. A total of 122 individuals were cleared to participate in the study and participated in a familiarization session. This included explanation of the protocol, instructions for completing the food record forms and training program logs, and practicing the strength and anaerobic capacity tests that were used in the study. Participants were then matched for age, body mass, and fat free mass (FFM) and randomized into one of three dietary supplement intervention arms in a randomized manner. A total of 80 males (22 ± 4 y, 178 ± 6 cm, 80.9 ± 13.9 kg, 15.2 ± 0.7% fat, 25.6 ± 4.0 kg/m^2^) completed the study.

### Strength training program

All participants were required to follow the same resistance training routine. The resistance training program consisted of training 4-days per week split into two upper and two lower body workouts per week primarily consisting of free-weight exercises for a total of 8-weeks. The 8-week training protocol was periodized in 2–3 week segments and consisted of selection from a list of 2–4 exercises for the following muscle groups: chest (two exercises for a total of six sets), back (two exercise for a total of six sets), shoulders (one exercise for a total of three sets), biceps (one exercise for a total of three sets), triceps (one exercise for a total of three sets), abdominals (one exercise for a total of three sets), quadriceps (two exercises for a total of six sets), hamstrings (two exercises for a total of six sets), and calves (one exercise for three sets). Each exercise consisted of three sets of ten repetitions (week 1–3), eight repetitions (week 4–6), or six repetitions (week 7–8) performed with as much weight as the participant could perform per set. The participants recorded the amount of weight lifted during each set of exercise on training log. A training partner or fitness instructor provided signed verification that the work out was completed as recorded. Prior research from our lab has shown that this program is effective in promoting significant strength and fat free mass gains in resistance-trained athletes without nutritional intervention [45].

### Supplementation protocol

Participants were matched for age, body mass, and FFM and randomly assigned to ingest in a double-blind manner either: (1) a flavored dextrose placebo (PLA); (2) a PWS containing beta-alanine (3 g), creatine nitrate as a salt (2 g), arginine alpha-ketoglutarate (2 g), N-Acetyl-L-Tyrosine (300 mg), caffeine (284 mg), Mucuna pruiriens extract standardized for 15% L-Dopa (15 mg), Vitamin C as Ascorbic Acid (500 mg), niacin (60 mg), folate as folic acid (50 mg), and 70 mg of Vitamin B12 as Methylcobalamin (*Cellucor C4 Pre-Workout, Nutrabolt, Bryan, TX*); or, 3.) the PWS with Citrus aurantium (PWS + S) extract standardized for 30% synephrine (20 mg) (*Nutratech Inc., Caldwell, NJ*). Supplements were independently packaged by a third party into coded single foil packets for double-blind administration following Good Manufacturing Practices and certified to contain the aforementioned ingredients by VMI Nutrition *(Salt Lake City, UT*). All supplements had similar color and powdered texture. Participants were instructed to ingest one foil packet per day approximately 15–30 min prior to exercise on training days and in the morning with breakfast on non-training days. Supplement compliance was verified by weekly compliance verification and collecting and counting empty packets.

### Testing sequence

**Fig. 1:**
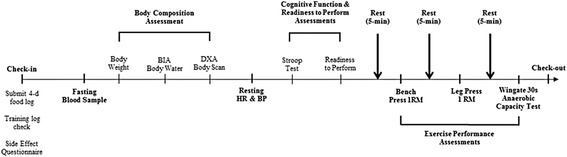
Study timeline

Figure 1 shows the timeline of tests performed. Participants were instructed to refrain from exercise, caffeine, and supplements containing stimulants for 48-h prior to testing. Participants presented to the lab after a 12-h fast and were required to provide a 4-days food-log that recorded their consumption of food and energy containing fluids. Participants training logs were assessed by a trained exercise physiologist to ensure compliance and they submitted weekly side-effect questionnaires. Participants then donated ~ 20 ml of blood via venipuncture. Following blood sampling, we administered a series of tests. This included determination of body weight, total body water using bioelectrical impedance (BIA), body composition using dual-energy x-ray absorptiometry (DXA), resting heart rate and blood pressure, cognitive function (Stroop Word - Color test), perceptions of readiness to perform via use of a visual analogue scale (VAS), one repetition maximum (1 RM) bench press, 1 RM leg press, and a 30s Wingate anaerobic capacity test on a cycle ergometer. Subjects rested 5-min between bench press, leg press, and Wingate tests as well as 2-min between sets on the bench press and leg press. Participants completed these assessments at 0, 4, and 8-weeks of training.

### Procedures

#### Training assessment

Total lifting volume was calculated based on information recorded on the training logs. This included multiplying the amount weight lifted per set times the number repetitions completed for each exercise performed during training sessions throughout the course of the study. Total lifting volume for upper and lower extremity lifts for the entire 8-week training period were calculated and analyzed to evaluate training volume.

#### Diet assessment

Participants were provided a detailed description of how to measure and record food and beverage intake on food logs by a registered dietitian. Participants recorded all food and energy containing fluids consumed for 4-days (including 1 weekend day) prior to each testing session. Food logs were checked for accuracy when returning to the lab for each testing session and entered and analyzed by a registered dietitian using dietary analysis software (*ESHA Food Processor Version 8.6, Salem, OR*).

#### Side effect assessment

A questionnaire developed in our lab and reported in numerous previously published papers [23, 46–49] was used to assess side effects in this study. Participants completed the survey every week throughout the study to determine how well participants tolerated supplementation; how well participants followed the supplementation protocol; and, if participants experienced any symptoms during the supplementation period. Subjects were asked to rank the frequency and severity of their symptoms for dizziness, headache, fast or racing heart rate, heart skipping or palpitations, shortness of breath, nervousness, blurred vision, and unusual or adverse effects. Additionally, participants ranked the frequency of symptoms with 0 (none), 1 (minimal: 1–2/week), 2 (slight: 3–4/week), 3 (occasional: 5–6/week), 4 (frequent: 7–8/week), or 5 (severe: 9 or more/week) as well as severity of symptoms with 0 (none), 1 (minimal), 2 (slight), 3 (moderate), 4 (severe), or 5 (very severe).

### Body composition

Body mass and height were determined according to standard procedures using a Healthometer Professional 500KL (*Pelstar LLC, Alsip, IL, USA)* self-calibrating digital scale with an accuracy of ± 0.02 kg. Total body water (TBW) was measured using bioelectrical impedance analysis (*ImpediMed DF50, San Diego, CA*) using standard procedures. Whole body bone density and body composition measures (excluding cranium) were determined with a Hologic Discovery W Dual-Energy X-ray Absorptiometer (DEXA; *Hologic Inc., Waltham, MA, USA*) equipped with APEX Software (*APEX Corporation Software, Pittsburg, PA, USA*) by using procedures previously described [8, 50]. Mean test-retest reliability studies performed on male athletes in our lab over repeated days revealed mean coefficients of variation (C_v_) for total bone mineral content and total fat free/soft tissue mass of 0.31–0.45% with a mean intraclass correlation of 0.985 [51]. On the day of each test, the equipment was calibrated following the manufacturer’s guidelines.

### Resting heart rate & blood pressure

As soon as the DXA scan was completed (about 6-min), resting heart rate was determined in the supine position by palpitation of the radial artery using standard procedures [52]. Blood pressure was then assessed by auscultation of the brachial artery using a mercurial sphygmomanometer using standard clinical procedures [52].

### Cognitive function assessment

Cognitive function was assessed using the Stroop Word-Color test standardized by Golden [53]. The test consists of three pages/tests with 100 items, presented in 5 columns of 20 items. Items on the first page (Word) are the color words RED, GREEN, and BLUE in black ink. On the second page (Color) the items are XXX’s colored in red, green, or blue ink. Items on the third page (Word-Color) are the words RED, GREEN, and BLUE printed in red, green, or blue ink with the limitation that word and ink could not match. Participants were given standardized instructions and asked to read aloud each word or color on each page as fast as they could for 45 s. The number of correct responses obtained on each test during the time period is used to assess cognitive function.

### Readiness to perform assessment

Perceptions about readiness to perform were assessed using a visual analogue scale (VAS) using a 5-item descriptive scale (strongly disagree, disagree, neutral, agree, strongly agree) arranged on a 20 cm dotted bar with these terms equidistant along the scale. Participants were asked to respond to the following questions; “*I slept well last night*”; “*I am looking forward to today’s workout*”; “*I am optimistic about my future performance*”; “*I feel vigorous and energetic*”; “*My appetite is great*”; and, “*I have little muscle soreness*”. Participants circled the number or dot between numbers that best described their current perceptions related to these questions.

### Strength testing

Strength tests were performed using an isotonic Olympic bench press (*Nebula Fitness, Versailles, OH*) according to standard procedures [45]. Participants followed a warm-up consisting of 10 repetitions using 50% of their estimated 1RM, 5 repetitions using 70% of their estimated 1RM, and 1 repetition using 90% of their estimated 1RM. Participants were given 2-min recovery between attempts and performed 1RM lifts until reaching a failure weight. After 5-min recovery, participants warmed-up in a similar fashion as described above and then performed 1RM lift attempts on a standard hip sled/leg press (*Nebula Fitness, Versailles, OH*) according to standard procedures [45]. Test to test reliability of performing these tests in our lab on resistance-trained participants have yielded low C_v_’s and high reliability for the bench press (1.9%, *r* = 0.94) and hip sled/leg press (0.7%, *r* = 0.91).

### Anaerobic capacity testing

Prior to performing the anaerobic capacity test, participants warmed-up on a bicycle ergometer at a self-selected work rate. Wingate anaerobic capacity tests were performed using a Lode Excalibur Sport Ergometer *(Lode BV, Groningen, The Netherlands*) with work rate set at of 7.5 J/kg/rev. Participants were asked to pedal as fast as possible prior to application of the workload and sprint at an all-out maximal capacity for 30s. This test measures absolute and relative peak and mean power and total work. Test-to-test variability in performing repeated Wingate anaerobic capacity tests in our laboratory yielded a C_v_ of 15% with a test retest correlation of *r* = 0.98 for mean power [47]. Participants practiced the anaerobic capacity test during the familiarization session to minimize learning effects.

### Blood chemistry

All blood samples were analyzed for standard blood chemistries inclusive of alkaline phosphatase (ALP), aspartate transaminase (AST), alanine transaminase (ALT), creatinine, blood urea nitrogen (BUN), creatine kinase (CK), lactate dehydrogenase (LDH), glucose, and blood lipids (total cholesterol, high density lipoprotein [HDL], low density lipoprotein [LDL], triglycerides [TG]) using a Cobas® c 111 (*Roche Diagnostics, Basel, Switzerland*). The internal quality control for the Cobas® c 111 was performed according to standard procedures [54] using two levels of control fluids purchased from the manufacturer to calibrate to acceptable SD’s and C_v_’s. Samples were re-run if the observed values were outside control values and/or clinical norms according to standard procedures. Test-to-test reliability assessment of assays evaluated in this study yielded mean C_V_’s < ±2.0% with *r* values > 0.99. We also assessed a complete blood count with platelet differential on whole blood (hemoglobin, hematocrit, red blood cell counts, mean corpuscle volume (MCV), mean corpuscle hemoglobin (MCH), mean corpuscle hemoglobin concentration (MCHC), red cell distribution width (RDW), white blood cell counts, lymphocytes, granulocytes, and mid-range absolute count (MID) using a Abbott Cell Dyn 1800 (*Abbott Laboratories, Abbott Park, IL, USA*) automated hematology analyzer. The internal quality control for Abbott Cell Dyn 1800 was performed using three levels of control fluids to calibrate to acceptable SD’s and C_v_’s. Test-to-test reliability assessment of assays evaluated in this study yielded mean C_V_’s < ±6.3% with *r* values > 0.9.

### Statistical analysis

Baseline demographic and training volume data were analyzed by one-way analysis of variance (ANOVA). All data were analyzed using general linear models (GLM) multivariate analysis of variance (MANOVA) with repeated measures with Wilks’ Lambda and Greenhouse-Geisser adjustments. For performance and cognitive function data, baseline values were used as a covariate and run with MANOVA for repeated measures with differences between groups assessed using a Dunnet-Hsu post-hoc assessment vs. the PLA condition. These data were also graphed with means and 95% Confidence Interval (CI) to determine whether changes from baseline were significant [55]. We also analyzed the number of changes in blood chemistry values observed from normal to exceeding normal clinical limits from baseline to week 4, baseline to week 8 and week 4 to week 8 using a Chi-square analysis to examine whether any nutritional treatment promoted a significant increase in the number of participants with values exceeding normal. All data are presented as mean ± SD or mean change and 95% CI.

## Results

### Participant demographics

Table 1 presents participant demographics by group assignment. A total of 80 participants completed the study (PLA = 27, PWS = 27, PWS + S = 26). One-way ANOVA revealed that no significant differences among groups in baseline age, height, body weight, or body mass index.

**Table 1:** Participant Demographics

Variable	Group	Number	Means ± SD	*p*-value
Age (y)	PLA	27	22.3 ± 3.9	0.31
	PWS	27	20.9 ± 3.9	
	PWS + S	26	22.0 ± 2.6	
Height (cm)	PLA	27	178.4 ± 6.9	0.64
	PWS	27	177.0 ± 4.6	
	PWS + S	26	177.8 ± 5.6	
Body Weight (kg)	PLA	27	81.1 ± 13.3	0.94
	PWS	27	81.5 ± 13.0	
	PWS + S	26	80.2 ± 15.8	
BMI (kg/m^2^)	PLA	27	25.4 ± 3.4	0.72
	PWS	27	26.1 ± 4.6	
	PWS + S	26	25.4 ± 3.4	

Values are means ± standard deviations. Variables were analyzed by one-way ANOVA

### Training volume and diet analysis

Table 2 presents total training volume observed among groups for upper and lower extremity exercises. One-way ANOVA analysis revealed that there were no significant differences in lifting volumes among groups. Table 3 shows 4-day diet analysis data observed among groups at 0, 4, and 8 weeks of training. MANOVA analysis revealed that no significant group x time interactions were observed among groups in relative energy intake (*p* = 0.19), protein intake (*p* = 0.72), carbohydrate intake (*p* = 0.55) or fat intake (*p* = 0.79).

**Table 2:** Total Training Volume Data

Variable	Group	Number	Total Volume	*p*-value
Upper body (kg)	PLA	25	279,831 ± 132,101	0.33
	PWS	27	236,691 ± 87,062	
	PWS + S	23	269,928 ± 103,130	
Lower body (kg)	PLA	25	314,516 ± 136,966	0.66
	PWS	27	283,825 ± 100,541	
	PWS + S	23	305,906 ± 133,611	

Values are means ± standard deviations. Total training volume was analyzed by one-way MANOVA. MANOVA analysis revealed overall Wilks’ Lambda group (*p* = 0.69). *p*-values reported are with between-subjects effects

**Table 3:** Dietary Analysis Data

Variable	Group	Number	Time (wk)				*p*-value
			0	4	8		
Energy Intake (kcal/d/kg)	PLA	26	27.06 ± 10.94	25.33 ± 9.31	24.72 ± 13.50	Group	0.27
	PWS	25	30.15 ± 8.76	25.72 ± 8.10	26.29 ± 10.99	Time	0.29
	PWS + S	23	29.03 ± 9.73	29.35 ± 13.75	32.13 ± 16.48	G x T	0.19
Protein (g/d/kg)	PLA	26	1.48 ± 0.57	1.46 ± 0.64	1.49 ± 0.79	Group	0.18
	PWS	25	1.54 ± 0.50	1.37 ± 0.47	1.51 ± 0.84	Time	0.86
	PWS + S	23	1.74 ± 0.79	1.83 ± 1.13	1.77 ± 0.94	G x T	0.72
Carbohydrate (g/d/kg)	PLA	26	2.60 ± 1.11	2.56 ± 0.95	2.21 ± 1.10	Group	0.91
	PWS	25	2.87 ± 1.10	2.46 ± 1.02	2.30 ± 1.00	Time	0.008
	PWS + S	23	2.61 ± 0.89	2.64 ± 1.43	2.42 ± 1.17	G x T	0.55
Fat (g/d/kg)	PLA	26	1.05 ± 0.54	0.90 ± 0.41	0.98 ± 0.67	Group	0.14
	PWS	25	1.22 ± 0.41	1.05 ± 0.45	1.14 ± 0.52	Time	0.30
	PWS + S	23	1.19 ± 0.51	1.19 ± 0.84	1.30 ± 0.71	G x T	0.79

Values are means ± standard deviations. Total calories, Protein, Carbohydrate, and Fat intake were analyzed by MANOVA. MANOVA analysis revealed overall Wilks’ Lambda group (*p* = 0.04), time (*p* = 0.03), and group x time (*p* = 0.35). Greenhouse-Geisser time and group x time (G x T) interaction *p*-values are reported with univariate group *p*-values

### Side effect analysis

Reported frequency and severity of dizziness, headaches, racing heart rate, palpitations, shortness of breath, nervousness, blurred vision, and/or other symptom were so infrequent among participants that statistical analysis was not valid as the vast majority of participants (i.e., 90–98%) typically reported 0 ratings on each item throughout the study. No study participant required medical referral.

### Body composition

Table 4 presents body composition data observed during the course of the study. MANOVA analysis revealed significant time effects in changes in body weight (0.96 ± 2.6 kg, *p* = 0.003) and FFM 0.67 ± 1.8 kg, *p* = 0.001). However, no significant interactions were observed among groups in body weight (*p* = 0.28), fat mass (*p* = 0.61), FFM (*p* = 0.28), body fat percentage (*p* = 0.36), or percent total body water (*p* = 0.37).

**Table 4:** Body Composition Data

Variables	Group	Number	Time (wk)				*p*-value
			0	4	8		
Body Weight (kg)	PLA	27	81.1 ± 13.4	81.8 ± 14.2	82.1 ± 14.0	Group	0.98
	PWS	27	81.8 ± 13.3	81.3 ± 11.3	82.2 ± 12.8	Time	0.003
	PWS + S	26	80.4 ± 16.1	81.3 ± 16.7	81.8 ± 17.3	G x T	0.28
Fat Mass (kg)	PLA	27	11.3 ± 5.4	11.9 ± 5.5	11.9 ± 5.2	Group	0.75
	PWS	27	12.7 ± 7.6	13.0 ± 7.7	12.8 ± 7.4	Time	0.10
	PWS + S	26	11.3 ± 7.3	11.6 ± 8.3	11.5 ± 8.5	G x T	0.61
Fat-Free Mass (kg)	PLA	27	63.0 ± 10.7	63.2 ± 11.2	63.4 ± 11.0	Group	0.94
	PWS	27	62.3 ± 7.2	62.2 ± 6.7	62.7 ± 6.6	Time	0.001
	PWS + S	26	62.4 ± 9.2	63.0 ± 9.0	63.6 ± 9.1	G x T	0.28
Body Fat (%)	PLA	27	15.1 ± 6.2	15.7 ± 6.0	15.7 ± 5.6	Group	0.55
	PWS	27	16.1 ± 6.6	16.4 ± 6.7	16.2 ± 6.3	Time	0.23
	PWS + S	26	14.5 ± 5.8	14.5 ± 6.3	14.3 ± 6.1	G x T	0.36
Total Body Water (%)	PLA	27	53.6 ± 6.4	53.0 ± 4.6	52.0 ± 5.5	Group	0.40
	PWS	27	50.5 ± 5.5	51.0 ± 5.7	51.7 ± 6.3	Time	0.82
	PWS + S	26	52.2 ± 5.7	51.4 ± 5.1	51.8 ± 5.9	G x T	0.37

Values are means ± standard deviations. All variables were analyzed by MANOVA. MANOVA analysis revealed overall Wilks’ Lambda group (*p* = 0.40), time (*p* = 0.003), and group x time (*p* = 0.50). Greenhouse-Geisser time and group x time (G x T) interaction *p*-values are reported with univariate group *p*-values

### Resting heart rate & blood pressure

MANOVA analysis revealed no significant differences among groups in hemodynamic responses during the study (Wilks’ Lambda group *p* = 0.62, time *p* = 0.33, and group x time *p* = 0.87). Univariate analysis revealed no indication that PWS or PWS + S supplementation increased resting heart rate (*p* = 0.26), systolic blood pressure (*p* = 0.96), or diastolic blood pressure (*p* = 0.54) during training compared to the PLA group. All group means remained within ± 2 beats/min for heart rate and ± 2 mmHg for blood pressure from baseline values.

### Cognitive function assessment

**Fig. 2:**
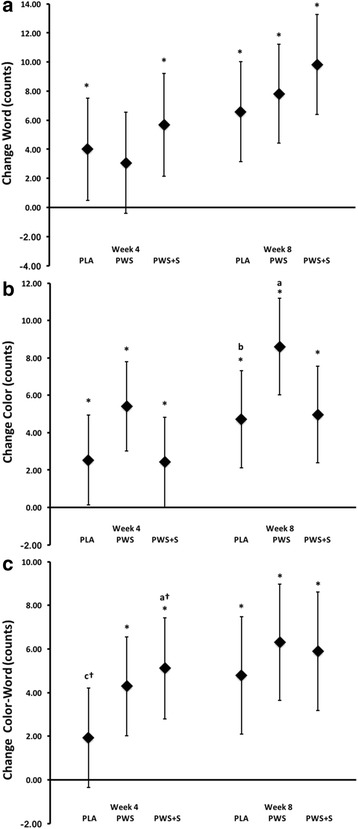
Changes in Stroop Word (Panel **a**), Color (Panel **b**), and Word-Color (Panel **c**) counts. Data are mean change and 95% CI

Table 5 shows the results for cognitive function testing. MANOVA analysis revealed Wilks’ Lambda overall time effects (*p* < 0.001) with no significant interaction effects (*p* = 0.17). MANOVA univariate analysis showed similar trends. However, univariate ANOVA analysis revealed an interaction trend (*p* = 0.087) among groups in color responses and a significant quadratic effect among groups in Word-Color counts (*p* = 0.04). Post-hoc analysis revealed that the PWS group demonstrated the greatest change in color counts from baseline while changes in word-color counts were seen sooner in the PWS and PWS + S groups (4-weeks) compared to the PLA group. Further, MANOVA analysis using baseline values as a covariate revealed some differences among groups in changes in cognitive function. As can be seen in Fig. 2, mean changes in Color, Word, and Word-Color counts where generally increased to a greater degree with 95% CI’s above baseline in the PWS and/or PWS + S groups compared to the PLA group values that were lower and had 95% CI’s crossing baseline. After 8-weeeks of intervention, all groups demonstrated significant increases in Color, Word, and Color-Word counts. More specifically, comparisons at week 4 demonstrated a significant increase in Word count for the PLA (3.92 counts, 95% CI .39, 7.45) and PWS + S (5.46 counts, 95% CI 2.09, 9.19) group, but not for PWS (3.21 counts, 95% CI −0.31, 6.72). By week 8, all groups increased their respective word counts: PLA (6.74 counts, 95% CI 3.32, 10.16), PWS (7.56 counts, 95% CI 4.15, 10.97) and PWS + S (9.93 counts, 95% CI 6.49, 13.73). For the Color assessment comparison, week 4 changes are: PLA (2.77 counts, 95% CI, 0.43, 5.09); PWS (5.05 counts, 95% CI, 2.72, 7.38); and, PWS + S (2.57 counts, 95% CI, 0.24, 4.88). For week 8, color assessment changes are: PLA (4.90 counts, 95% CI, 2.32, 7.46); PWS (8.33 counts, 95% CI, 5.76, 10.89); and, PWS + S (5.08 counts, 95% CI, 2.51, 7.63). Week 4 word-color changes were significant for the PWS (3.99 counts, 95% CI, 1.75, 6.23), and PWS + S (5.27 counts, 95% CI, 3.01, 7.52), but not the PLA (2.08 counts, 95% CI, −0.15, 4.31) group. By week 8, all groups demonstrated a significant increase in word-color counts: PLA (5.02 counts, 95% CI, 2.44, 7.59); PWS (5.84 counts, 95% CI, 3.27, 8.41); and, PWS + S (6.13 counts, 95% CI, 3.54, 8.72).

**Table 5:** Stroop Word-Color Cognitive Function Data

Variable	Group	Number	Time (wk)				*p*-value
			0	4	8		
Word (counts)	PLA	27	109.8 ± 16.9	113.6 ± 17.2	116.2 ± 17.5	Group	0.33
	PWS	27	107.7 ± 11.4	110.6 ± 10.9	115.3 ± 10.9	Time	<0.001
	PWS + S	26	102.3 ± 11.2	108.2 ± 17.0	112.4 ± 16.1	G x T	0.45
	Mean ± SD		106.7 ± 13.7	110.8 ± 15.3*	114.7 ± 15.0*^		
Color (counts)	PLA	27	80.8 ± 10.6^c^	83.2 ± 11.8^c^	85.7 ± 12.5*^c^	Group	0.35
	PWS	27	78.2 ± 9.4	83.6 ± 9.6*^c^	86.7 ± 10.1*^^c^	Time	<0.001
	PWS + S	26	77.0 ± 10.4^a^	79.7 ± 9.8*^ab^	82.1 ± 10.8*^ab^	G x T	0.087_l_
	Mean ± SD		78.7 ± 10.1	82.2 ± 10.5*	84.9 ± 11.2*^		
Word-Color (counts)	PLA	27	54.1 ± 11.5^c^	55.5 ± 11.5	58.5 ± 12.6*^c^	Group	0.42
	PWS	27	53.1 ± 5.9^c^	56.7 ± 7.4*	59.1 ± 7.6*^c^	Time	<0.001
	PWS + S	26	49.2 ± 11.1^ab^	55.0 ± 9.6*	55.6 ± 10.2^*ab^	G x T	0.04_q_

Values are means ± standard deviations. Word, Color, and Word-Color counts were analyzed by MANOVA. MANOVA analysis revealed overall Wilks’ Lambda group (*p* = 0.83), time (*p* < 0.001), and group x time (*p* = 0.17). Greenhouse-Geisser time and group x time (G x T) interaction *p*-values are reported with univariate group *p*-values. _q_ represents quadratic effect and _l_ represents linear *p*-value from univariate ANOVA. * represents *p* < 0.05 difference from baseline. ^ represents *p* < 0.05 difference from wk 4. ^a^represents *p* < 0.05 from PLA. ^b^represents *p* < 0.05 from PWS. ^c^represents *p* < 0.05 from PWS + S

### Readiness to perform assessment

Table 6 presents Readiness to Perform VAS data observed throughout the study. MANOVA revealed an overall time effect with no significant interactions among groups. Likewise, MANOVA using baseline values and age as a covariate and analysis of mean changes with 95% CI’s revealed no significant differences among groups.

**Table 6:** Readiness to Perform Visual Analogue Scale Data

Variable	Group	Number	Time (wk)				*p*-value
			0	4	8		
I slept well last night	PLA	27	3.46 ± 0.90	3.43 ± 0.92	3.53 ± 0.90	Group	0.25
	PWS	27	3.77 ± 0.93^a^	3.75 ± 0.89	3.52 ± 1.17	Time	0.83
	PWS + S	26	3.21 ± 1.09^b^	3.26 ± 1.11	3.59 ± 0.91	G x T	0.34
I am looking forward to today’s workout	PLA	27	3.93 ± 0.64	3.92 ± 0.61	3.63 ± 0.84	Group	0.94
	PWS	27	3.85 ± 0.90	4.01 ± 0.68	3.77 ± 0.86	Time	0.22
	PWS + S	26	3.73 ± 0.66	3.92 ± 0.68	3.93 ± 0.82	G x T	0.35
I am optimistic about my future performance	PLA	27	4.39 ± 0.49	4.16 ± 0.66	3.71 ± 0.95	Group	0.25
	PWS	27	4.50 ± 0.63	4.42 ± 0.63	4.05 ± 0.66	Time	<0.001
	PWS + S	26	4.21 ± 0.75	4.30 ± 0.67	3.88 ± 0.99	G x T	0.51
I feel vigorous and energetic	PLA	27	3.56 ± 0.71	3.24 ± 0.75	3.21 ± 0.93	Group	0.26
	PWS	27	3.46 ± 0.94	3.46 ± 0.84^a^	3.55 ± 0.73	Time	0.17
	PWS + S	26	3.21 ± 0.80	3.00 ± 0.93^b^	3.38 ± 0.85	G x T	0.17
My appetite is great	PLA	27	4.48 ± 0.64^b^	4.25 ± 0.81	4.17 ± 0.79	Group	0.13
	PWS	27	4.03 ± 0.81^c^	4.03 ± 0.88	4.03 ± 0.79	Time	0.12
	PWS + S	26	4.05 ± 0.92	3.96 ± 1.03	3.73 ± 1.11	G x T	0.63
I have little muscle soreness	PLA	27	3.84 ± 0.73	3.84 ± 0.99	3.58 ± 1.04	Group	0.66
	PWS	27	3.56 ± 1.15	3.67 ± 1.11	3.52 ± 1.00	Time	0.93
	PWS + S	26	3.52 ± 1.17	3.52 ± 1.17	3.73 ± 1.11	G x T	0.68

Values are means ± standard deviations. Six questions were analyzed by MANOVA. MANOVA analysis revealed overall Wilks’ Lambda group (*p* = 0.27), time (*p* < 0.001), and group x time (*p* = 0.66). Greenhouse-Geisser time and group x time (G x T) interaction *p*-values are reported with univariate group *p*-values. * represents *p* < 0.05 difference from baseline. ^ represents *p* < 0.05 difference from wk 4. ^a^represents *p* < 0.05 from PLA. ^b^represents *p* < 0.05 from PWS. ^c^represents *p* < 0.05 from PWS + S

### Performance assessment

**Fig. 3:**
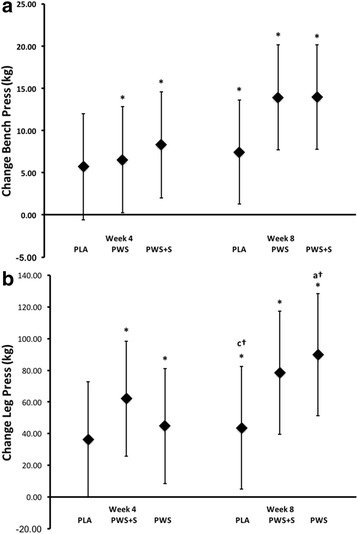
Changes in 1RM bench press (Panel **a**) and 1RM leg press (Panel **b**). Data are mean change and 95% CI

Performance testing outcomes are presented in Table 7. MANOVA revealed significant time effects for bench press and leg press 1RM performance. However, no significant univariate interactions were observed among groups. MANOVA analysis using baseline values as a covariate and assessment of mean change and 95% CI’s of 1RM strength data (Fig. 3) revealed that there were significant increases in bench press 1RM strength at week 4 for the PWS (8.17 kg; 95% CI 1.88, 14.47) and PWS + S (6.95 kg; 95% CI 0.62, 13.28), but not for the PLA (5.45 kg, 95% CI −0.82, 11.73). By week 8, all groups demonstrated a significant increase in BP 1 RM: PLA (7.18 kg, 95% CI 1.01, 13.36), PWS (14.36, 95% CI 8.13, 20.59) and PWS + S (13.84 kg, 95% CI 7.64, 20.04). No between group differences were noted at week 4 or week 8. A similar pattern for leg press 1 RM strength improvement was observed within the PWS (61.84 kg, 95% CI 24.96, 98.725) and PWS + S 44.89 kg, 95% CI 8.31, 81.47) groups, but not for PLA (36.50, 95% CI, −0-21, 73.2). Similarly, by week 8, all groups increased their LP 1RM as follows: PLA (43.28 kg, 95% CI 4.16, 82.41), PWS (79.23 kg, 95% CI 39.12, 118.54), and PWS + S (89.54 kg, 95% CI 50.55, 128.53). No between groups differences were noted at week 4 or week 8. Similar results were observed in Wingate anaerobic capacity assessment. MANOVA analysis using baseline values and ages as covariates revealed that a significant increase in Wingate peak power for PWS + S and PLA at week 4; yet no significant differences were otherwise noted at week 8 or among other Wingate parameters examined.

**Table 7:** Performance Data

Variable	Group	Number	Time (wk)				*p*-value
			0	4	8		
Bench Press (kg)	PLA	27	100.6 ± 20.8	102.9 ± 24.7	104.0 ± 23.3	Group	0.56
	PWS	27	96.7 ± 23.1	99.2 ± 21.3	102.7 ± 21.7	Time	<0.001
	PWS + S	26	102.0 ± 16.3	106.2 ± 16.7	108.6 ± 17.1	G x T	0.33
Leg Press (kg)	PLA	27	472.8 ± 149.8	490.3 ± 134.2	491.1 ± 131.0	Group	0.67
	PWS	27	436.6 ± 96.6	466.0 ± 101.9	474.0 ± 96.3	Time	<0.001
	PWS + S	26	454.2 ± 79.4	474.4 ± 92.1	494.9 ± 100.9	G x T	0.28
Peak Power (Watt)	PLA	27	1,472 ± 485	1,630 ± 475	1,507 ± 385	Group	0.91
	PWS	27	1,502 ± 384	1,602 ± 354	1,603 ± 466	Time	0.01
	PWS + S	26	1,445 ± 324	1,605 ± 444	1,544 ± 403	G x T	0.83
Mean Power (Watt)	PLA	27	647 ± 146	640 ± 133	636 ± 127	Group	0.95
	PWS	27	630 ± 72	643 ± 66	638 ± 69	Time	0.23
	PWS + S	26	630 ± 114	656 ± 134	612 ± 145	G x T	0.40
Peak Power (Watt/kg)	PLA	27	18.3 ± 5.6	20.2 ± 5.4	18.4 ± 4.0	Group	0.90
	PWS	27	18.6 ± 4.7	19.8 ± 4.6	19.8 ± 5.8	Time	0.02
	PWS + S	26	18.3 ± 3.6	20.2 ± 5.3	19.4 ± 4.8	G x T	0.80
Mean Power (Watt/kg)	PLA	27	8.0 ± 1.1	7.9 ± 1.0	7.7 ± 0.9	Group	0.73
	PWS	27	7.8 ± 1.2	8.0 ± 1.1	7.9 ± 1.0	Time	0.18
	PWS + S	26	8.0 ± 1.1	8.2 ± 1.4	8.0 ± 1.2	G x T	0.33
Total Work (Joules)	PLA	27	19,394 ± 4,377	19,217 ± 4,019	19,109 ± 3,826	Group	0.98
	PWS	27	18,927 ± 2,166	19,319 ± 2,003	19,156 ± 2,095	Time	0.17
	PWS + S	26	18,923 ± 3,431	19,680 ± 4,025	19,213 ± 3,110	G x T	0.29

Values are means ± standard deviations. Strength (Bench and Leg Press) were analyzed by MANOVA. MANOVA analysis revealed overall Wilks’ Lambda group (*p* = 0.58), time (*p* < 0.001), and group x time (*p* = 0.29). Greenhouse-Geisser time and group x time interaction *p*-values are reported with univariate group *p*-values. * represents *p* < 0.05 difference from baseline. ^ represents *p* < 0.05 difference from wk 4

### Blood chemistry assessment

Tables 8, 9 and 10 show blood chemistry data analyzed during the study. MANOVA revealed some time effects in several variables indicative of individuals engaged in heavy resistance exercise training with no significant group x time interactions in muscle and liver enzymes, markers of catabolism, or blood lipids. Univariate ANOVA analysis revealed a significant quadratic effect in blood glucose values. Post-hoc analysis revealed the PLA group had a small but significant increase in blood glucose after 4-weeks of training while all groups were higher after 8-weeks of training. However, no significant differences were seen among groups and values remained within normal ranges. Table 11 shows Chi squared categorical analysis. No significant differences were observed among groups in the number of participants who observed changes in blood chemistry markers above normal baseline values.

**Table 8:** Muscle and Liver enzymes and Markers of Catabolism

Variable	Group	Number	Time (wk)				*p*-value
			0	4	8		
ALP (U/L)	PLA	27	76.8 ± 15.1	66.4 ± 12.9	71.2 ± 13.4	Group	0.24
	PWS	27	87.2 ± 24.7	72.3 ± 19.0	78.4 ± 24.9	Time	<0.001
	PWS + S	26	76.4 ± 22.8	68.1 ± 17.3	74.4 ± 19.6	G x T	0.25
ALT (U/L)	PLA	27	25.3 ± 10.0	25.0 ± 22.4	24.6 ± 13.0	Group	0.74
	PWS	27	25.1 ± 10.1	26.9 ± 16.0	30.9 ± 17.9	Time	0.73
	PWS + S	26	26.6 ± 23.0	28.6 ± 20.8	26.0 ± 12.2	G x T	0.55
AST (U/L)	PLA	27	29.1 ± 11.0	27.6 ± 17.7	27.1 ± 9.8	Group	0.47
	PWS	27	28.8 ± 9.7	31.6 ± 26.1	33.1 ± 19.7	Time	0.96
	PWS + S	26	28.4 ± 15.1	28.4 ± 10.3	27.3 ± 8.5	G x T	0.76
CK (U/L)	PLA	27	310.0 ± 217.2	263.1 ± 214.4	215.5 ± 115.1	Group	0.34
	PWS	27	297.5 ± 160.4	256.9 ± 203.7	336.1 ± 242.0	Time	0.79
	PWS + S	26	261.2 ± 130.0	324.1 ± 232.2	274.8 ± 172.3	G x T	0.86
LDH (U/L)	PLA	27	164.2 ± 36.2	153.5 ± 33.9	159.4 ± 29.5	Group	0.90
	PWS	27	157.7 ± 24.2	160.7 ± 37.6	162.8 ± 24.3	Time	0.19
	PWS + S	26	158.8 ± 32.1	152.5 ± 24.3	160.4 ± 23.6	G x T	0.44
BUN (mg/dl)	PLA	27	14.9 ± 4.9 ^b^	12.3 ± 6.7	16.2 ± 6.8	Group	0.62
	PWS	27	12.0 ± 4.0 ^c^	12.3 ± 5.8	16.1 ± 5.3	Time	<0.001
	PWS + S	26	13.6 ± 4.9	12.6 ± 6.6	16.5 ± 5.0	G x T	0.55
Creatinine (mg/dl)	PLA	27	1.15 ± 0.44	0.72 ± 0.47	0.85 ± 0.36^a^	Group	0.27
	PWS	27	1.08 ± 0.41	0.75 ± 0.38	0.95 ± 0.31	Time	<0.001
	PWS + S	26	1.13 ± 0.51	0.82 ± 0.43	1.05 ± 0.28^c^	G x T	0.78
BUN:Creatinine	PLA	27	15.7 ± 9.6	24.7 ± 19.1	26.5 ± 23.4	Group	0.20
	PWS	27	12.7 ± 6.8	22.7 ± 17.3	20.4 ± 15.0	Time	0.002
	PWS + S	26	15.3 ± 10.9	20.9 ± 15.1	17.9 ± 12.6	G x T	0.63

Values are means ± standard deviations. All variables were analyzed by MANOVA. MANOVA analysis revealed overall Wilks’ Lambda group (*p* = 0.43), time (*p* < 0.001), and group x time (*p* = 0.66). Greenhouse-Geisser time and group x time (G x T) interaction *p*-values are reported with univariate group *p*-values. * represents *p* < 0.05 difference from baseline. ^ represents *p* < 0.05 difference from wk 4

**Table 9:** Blood Glucose and Lipid Data

Variable	Group	Number	Time (wk)				*p*-value
			0	4	8		
Glucose (mg/dl)	PLA	27	86.6 ± 7.0	93.7 ± 12.8*	93.8 ± 13.0*	Group	0.90
	PWS	27	89.4 ± 7.1	89.7 ± 8.9	95.1 ± 9.2*	Time	<0.001
	PWS + S	26	90.7 ± 8.2	90.1 ± 5.0	95.4 ± 7.9*	G x T	0.017_q_
Cholesterol (mg/dl)	PLA	27	159.5 ± 31.6	156.9 ± 34.3	156.1 ± 41.7	Group	0.34
	PWS	27	164.1 ± 31.3	158.1 ± 26.0	163.3 ± 32.8	Time	0.16
	PWS + S	26	174.5 ± 42.8	171.9 ± 40.9	165.9 ± 36.1	G x T	0.44
HDL-C (mg/dl)	PLA	27	53.4 ± 12.6	51.8 ± 13.1	51.3 ± 15.0	Group	0.99
	PWS	27	52.7 ± 14.7	50.0 ± 13.9	52.6 ± 14.7	Time	0.10
	PWS + S	26	53.5 ± 16.2	52.7 ± 12.7	49.6 ± 15.0	G x T	0.24
CHL:HDL	PLA	27	3.1 ± 0.7	3.1 ± 0.8	3.1 ± 0.7	Group	0.38
	PWS	27	3.2 ± 0.9	3.3 ± 0.8	3.2 ± 0.7	Time	0.35
	PWS + S	26	3.9 ± 3.7	3.5 ± 1.8	4.1 ± 4.4	G x T	0.22
LDL-C (mg/dl)	PLA	27	106.0 ± 29.6	105.0 ± 31.4	104.7 ± 35.1	Group	0.32
	PWS	27	111.4 ± 29.8	108.0 ± 23.4	110.6 ± 27.9	Time	0.43
	PWS + S	26	121.0 ± 35.2	119.2 ± 42.3	116.2 ± 42.2	G x T	0.56
Triglyceride (mg/dl)	PLA	27	74.2 ± 33.8	81.9 ± 31.5	83.6 ± 30.7	Group	0.11
	PWS	27	92.8 ± 43.3	98.5 ± 37.8^a^	86.5 ± 37.6	Time	0.89
	PWS + S	26	81.6 ± 36.6	73.9 ± 22.9^b^	80.4 ± 41.9	G x T	0.31

Values are means ± standard deviations. All variables were analyzed by MANOVA. MANOVA analysis revealed overall Wilks’ Lambda group (*p* = 0.44), time (*p* < 0.001), and group x time (*p* = 0.58). Greenhouse-Geisser time and group x time (G x T) interaction *p*-values are reported with univariate group *p*-values. _q_ represents quadratic effect and _l_ represents linear *p*-value from univariate ANOVA. * represents *p* < 0.05 difference from baseline. ^ represents *p* < 0.05 difference from wk 4

**Table 10:** Complete Blood Count Data

Variable	Group	Number	Time (wk)				*p*-value
			0	4	8		
WBC Count (x10^3^/μl)	PLA	27	6.09 ± 2.09	5.84 ± 1.17	6.04 ± 1.52	Group	0.36
	PWS	27	6.64 ± 1.54	6.21 ± 1.66	5.92 ± 1.17	Time	0.09
	PWS + S	26	5.98 ± 1.25	5.66 ± 1.40	5.68 ± 1.74	G x T	0.64
RBC Count (x10^6^/μl)	PLA	27	5.09 ± 0.72	4.82 ± 0.47	4.87 ± 0.54	Group	0.72
	PWS	27	4.92 ± 0.51	4.92 ± 0.51	4.88 ± 0.34	Time	0.88
	PWS + S	26	4.87 ± 0.60	5.04 ± 0.56	5.05 ± 0.64	G x T	0.16
Hemoglobin (g/dl)	PLA	27	15.55 ± 2.49	14.90 ± 1.35	15.12 ± 1.46	Group	0.84
	PWS	27	15.24 ± 1.33	15.26 ± 1.76	15.03 ± 0.95	Time	0.97
	PWS + S	26	14.84 ± 2.13	15.60 ± 1.78	15.61 ± 1.98	G x T	0.13
Hematocrit (%)	PLA	27	47.28 ± 6.86	44.66 ± 4.31	45.04 ± 5.08	Group	0.76
	PWS	27	45.84 ± 4.71	45.74 ± 5.60	45.45 ± 3.07	Time	0.85
	PWS + S	26	45.24 ± 5.77	46.87 ± 5.22	46.77 ± 5.77	G x T	0.16
MCV (fL)	PLA	27	92.87 ± 3.89	92.72 ± 3.51	92.48 ± 3.61	Group	0.34
	PWS	27	93.22 ± 3.37	92.98 ± 3.28	93.18 ± 2.86	Time	0.32
	PWS + S	26	92.74 ± 3.03	92.98 ± 3.26	92.60 ± 3.27	G x T	0.37
MCH (pg/cell)	PLA	27	30.53 ± 1.76	30.98 ± 2.09	31.12 ± 1.81	Group	0.80
	PWS	27	31.06 ± 1.44	31.07 ± 1.53	30.83 ± 1.48	Time	0.13
	PWS + S	26	30.37 ± 1.33	30.96 ± 1.24	30.93 ± 1.60	G x T	0.36
MCHC (g/dl)	PLA	27	32.87 ± 1.39	33.40 ± 1.46	33.69 ± 1.90	Group	0.73
	PWS	27	33.34 ± 1.35	33.41 ± 1.42	33.09 ± 1.22	Time	0.11
	PWS + S	26	32.75 ± 0.91	33.31 ± 1.37	33.39 ± 1.10	G x T	0.30
RDW (%)	PLA	27	13.38 ± 0.62^c^	13.28 ± 0.51	12.98 ± 0.59	Group	0.43
	PWS	27	13.09 ± 0.58	13.11 ± 0.88	13.18 ± 0.73	Time	0.65
	PWS + S	26	12.97 ± 0.58^a^	12.99 ± 0.77	13.08 ± 0.72	G x T	0.02
Platelet Count (x10^3^/μl)	PLA	27	220.66 ± 75.84	233.14 ± 53.34	228.96 ± 55.60	Group	0.46
	PWS	27	230.77 ± 45.80	234.11 ± 38.24	242.92 ± 41.94	Time	0.58
	PWS + S	26	222.53 ± 67.23	222.26 ± 48.51	217.38 ± 51.95	G x T	0.65

Values are means ± standard deviations. All variables were analyzed by MANOVA. MANOVA analysis revealed overall Wilks’ Lambda group (*p* = 0.78), time (*p* = 0.06), and group x time (*p* = 0.013). Greenhouse-Geisser time and group x time (G x T) interaction *p*-values are reported with univariate group *p*-values. ^a^represents *p* < 0.05 from PLA. ^b^represents *p* < 0.05 from PWS. ^c^represents *p* < 0.05 from PWS + S

**Table 11:** Prevalence of Blood Chemistry Changes Exceeding Normal Clinical Bounds

	Marker	Category	PLA	PWS	PWS + S	*p*-value
Lipids & Glucose	Cholesterol	No Change	23	24	25	0.42
		Normal Baseline, Exceed at 4-weeks	2	0	1	
		Normal Baseline, Exceed at 8-weeks	0	1	0	
		Normal 4-weeks, Exceed 8-weeks	2	2	0	
	HDL-C	No Change	25	21	26	0.10
		Normal Baseline, Exceed at 4-weeks	1	1	0	
		Normal Baseline, Exceed at 8-weeks	1	1	0	
		Normal 4-weeks, Exceed 8-weeks	0	4	0	
	LDL-C	No Change	15	15	17	0.49
		Normal Baseline, Exceed at 4-weeks	11	9	9	
		Normal Baseline, Exceed at 8-weeks	1	1	0	
		Normal 4-weeks, Exceed 8-weeks	0	2	0	
	Triglycerides	No Change	27	27	25	0.35
		Normal Baseline, Exceed at 4-weeks	0	0	1	
		Normal Baseline, Exceed at 8-weeks	0	0	0	
		Normal 4-weeks, Exceed 8-weeks	0	0	1	
	Glucose	No Change	21	23	24	0.49
		Normal Baseline, Exceed at 4-weeks	2	0	0	
		Normal Baseline, Exceed at 8-weeks	1	1	0	
		Normal 4-weeks, Exceed 8-weeks	3	3	2	
Muscle	LDH	No Change	20	18	18	0.74
		Normal Baseline, Exceed at 4-weeks	1	2	0	
		Normal Baseline, Exceed at 8-weeks	2	1	3	
		Normal 4-weeks, Exceed 8-weeks	4	6	5	
	Creatine Kinase	No Change	19	22	21	0.42
		Normal Baseline, Exceed at 4-weeks	1	0	0	
		Normal Baseline, Exceed at 8-weeks	0	1	2	
		Normal 4-weeks, Exceed 8-weeks	7	4	3	
Kidney	Creatinine	No Change	23	25	23	0.72
		Normal Baseline, Exceed at 4-weeks	2	0	1	
		Normal Baseline, Exceed at 8-weeks	0	0	0	
		Normal 4-weeks, Exceed 8-weeks	2	2	2	
	BUN	No Change	17	18	19	0.41
		Normal Baseline, Exceed at 4-weeks	3	2	1	
		Normal Baseline, Exceed at 8-weeks	0	0	2	
		Normal 4-weeks, Exceed 8-weeks	7	7	4	
Liver	ALP	No Change	25	23	25	0.28
		Normal Baseline, Exceed at 4-weeks	1	0	0	
		Normal Baseline, Exceed at 8-weeks	0	0	0	
		Normal 4-weeks, Exceed 8-weeks	1	4	1	
	ALT	No Change	24	22	22	0.72
		Normal Baseline, Exceed at 4-weeks	1	0	0	
		Normal Baseline, Exceed at 8-weeks	1	2	1	
		Normal 4-weeks, Exceed 8-weeks	1	3	3	
	AST	No Change	23	21	21	0.84
		Normal Baseline, Exceed at 4-weeks	1	1	1	
		Normal Baseline, Exceed at 8-weeks	0	1	2	
		Normal 4-weeks, Exceed 8-weeks	3	4	2	

Data are frequency of occurrence. Significance is by chi-square analysis

## Discussion

Numerous PWS’s are sold to athletes purporting to improve acute exercise performance and/or promote greater training adaptations [1]. Preliminary assessment of the acute effects of ingesting the PWS used in this study provided some evidence that this formulation enhanced resting energy expenditure and cognitive function and that adding 20 mg of synephrine to the formulation increased resting energy expenditure to a greater degree [42, 43]. Theoretically, use of these PWS’s during training would promote greater fat loss and/or training adaptations over time. Therefore, this study examined the safety and efficacy of daily ingestion of a commercially available PWS with and without synephrine for 8-weeks during training on body composition and training adaptations in resistance-trained athletes. Results indicated that while there was some evidence that PWS ingestion enhanced some measures of cognitive function and 1RM strength gains primarily after 4-weeks of training, no significant differences were seen among groups in improvement in body composition and/or cognitive and exercise performance after 8-weeks of training. Additionally, there was little evidence indicating that adding synephrine to the PWS promoted additive benefits. Results also indicated that ingesting these PWS’s had no significant effects on resting heart rate or blood pressure, standard clinical chemistry panels, and/or the incidence of reported side effects. Consequently, within the confines of this study, use of these PWS’s appeared to be well-tolerated.

### Cognitive function

The PWS’s investigated in this study contained 284 mg of caffeine, 300 mg of N-Acetyl-L-Tyrosine, and 15 mg of L-Dopa extracted from Mucuna pruiriens. Numerous studies have shown that ingesting caffeine (e.g., 3–6 mg/kg) can improve exercise performance, cognitive function, and/or vigilance [5]. In the present study, participants ingested an average of 3.5 mg/kg of caffeine prior to training sessions. There is also some evidence that tyrosine supplementation can improve cognitive function and/or memory [13, 14] although dosages typically studied (i.e., 50–150 mg/kg or 1.6–12 g) were much higher than the amount investigated in this study (i.e., 300 mg). Additionally, there is some evidence that L-Dopa supplementation (e.g., 150 mg) can affect cognitive function and/or reduce perceptions of fear or anxiety [15, 16] although the dosages studied in the present investigation are much lower. Thus, it is conceivable that ingesting a PWS with ergogenic levels of caffeine (and possibly tyrosine and/or L-Dopa) prior to exercise may provide some improvement in cognitive function and/or training adaptations due to a greater ability to focus on exercise training.

In the present study, analysis of mean changes from baseline with 95% CI’s (Fig. 2) indicated that change in Stroop Color and Word-Color counts were significantly above baseline in the PWS group and changes in Word count, Color count, and Word-Color counts were greater in the PWS + S group after 4-weeks of training and these changes were generally greater than the placebo group. These findings are consistent with several studies that reported that acute or chronic ingestion of PWS formulations containing caffeine positively affected cognitive function, concentration, and/or perceptions of energy [5, 56, 57]. The Stroop Color-Word test assesses attention, processing speed, and executive function which have been shown to decline as one fatigues. Theoretically, maintaining cognitive function during the latter stages of competition can improve performance particularly in events that require quick decision making. However, after 8-weeks of training and supplementation in the present study, all groups observed similar improvements in Stroop Word, Color, and Word-Color counts and no differences were observed in questions related to readiness to perform. These findings are consistent with other studies reporting no overall benefit of PWS supplementation on cognitive function, concentration, and/or perceptions of energy [58, 59] and suggest that the ergogenic value observed may lessen over time. However, it is also possible that since performing the 1 RM and anaerobic sprint capacity tests did not require significant executive or cognitive functioning to perform, there may be benefits on other performance tasks that require more decision making and attention.

### Training adaptations

The PWS’s examined in the present study contained a number of nutrients that have been purported to influence training adaptations. For example, the PWS’s contained 3 g/day of beta-alanine. Beta-alanine supplementation (e.g., 3–6 g/day for 2–4 weeks) has been reported to increase muscle carnosine levels, buffer acidity, and enhance exercise performance [6, 21–24]. Research has indicated that length of time of supplementation affects the impact that beta-alanine supplementation has on muscle carnosine levels and potential ergogenic benefit [27, 60–62]. Thus, it is possible that consuming 3 g/day of beta-alanine for 8-weeks could have provided ergogenic benefit during resistance-training. The PWS supplements also contained 2 g/day of creatine nitrate at approximately a 2:1 ratio of creatine to nitrate. While there is little evidence that ingesting 2 g/day of creatine for 8-weeks can markedly increase muscle creatine stores and/or affect exercise capacity, it is enough creatine to maintain creatine stores during training [7, 28]. The greater potential of ergogenic value from ingesting 2 g/day of creatine nitrate would theoretically be due to the nitrate content [46, 63]. There are a number of studies that indicate that nitrate supplementation prior to exercise (e.g., 300 mg) can improve exercise capacity [9, 11, 17–20]. The amount of nitrate contained in the creatine nitrate supplement used (i.e., ~1 g/day) is more consistent with dietary recommendations to help control blood pressure [64, 65]. Nevertheless, there was sufficient amount of nitrates in the PWS to provide some ergogenic benefit particularly to endurance exercise performance. The PWS’s also contained 2 g/day of arginine alpha-ketoglutarate. Prior research has reported that dietary supplementation of higher doses of arginine alpha keto-glutarate (e.g., 12 g/day) can affect exercise capacity [12] although other studies with lower amounts have shown no benefit [66, 67]. Given that the PWS’s only contained 2 g/day of arginine alpha-ketoglutarate, it is likely that this nutrient provided minimal effects. Finally, one of the PWS’s contained 20 mg of synephrine (as *Citrus Aurantium*). Synephrine has been purported to suppress appetite [29] and promote weight loss through an enhanced thermogenesis [34–36] with no negative effects on the cardiovascular system [37, 38, 68]. Theoretically, adding synephrine to the PWS would promote greater fat loss during training and a more optimal body composition.

**Fig. 4:**
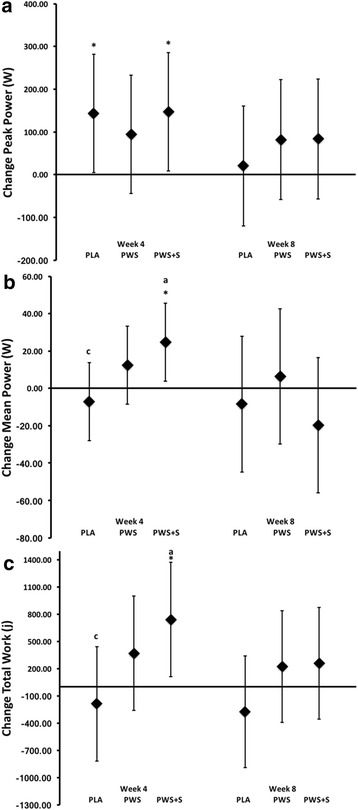
Changes in Wingate anaerobic capacity test peak power (Panel **a**), mean power (Panel **b**), and total work (Panel **c**). Data are mean change and 95% CI

Results of the present study provide some support that ingesting a PWS containing these nutrients enhanced training adaptations. Analysis of mean changes from baseline and 95% CI data (see Fig. 4) indicated that there was some evidence that 1RM strength gains were greater with PWS supplementation compared to placebo at both 4 and 8-weeks of training. There was also evidence that peak power, mean power, and total work during the anaerobic capacity sprint test was increased to a greater degree after 4-weeks of PWS + S supplementation (see Fig. 4). However, consistent with findings from Outlaw and colleagues [58], we did not observe a significant improvement in body composition changes in response to training as has previously been reported [56, 69]. This includes when adding 20 mg of synephrine (as Citrus Aurantium) to the formulation. These latter findings contrast reports suggesting that that Citrus Aurantium supplementation can promote weight and/or fat loss [36, 70] although it should be noted that the amount of synephrine provided (i.e., 20 mg/day) was less than that used in Kaats et al. study (i.e., 49 or 98 mg/day). Greenway and coworkers [71] reported that ingesting an herbal supplement containing 200 mg of green tea, 198 mg of caffeine (derived from guarana extract), and 9 mg of synephrine (derived from Citrus Aurantium) did not promote weight loss. Thus, it is possible that a higher dose of synephrine is needed to promote weight loss in individuals involved in intense training and/or addition of synephrine to a PWS has less effect in resistance-trained individuals.

### Safety analysis

One of the criticisms regarding PWS’s is that while there may be some ergogenic benefit, the short and/or long-term safety of taking these supplements is unknown [1]. Results of the present investigation indicate that the PWS’s studied were well-tolerated and did not adversely affect markers of health. In this regard, we observed no significant changes in resting hemodynamics, clinical blood markers, or a disproportional number of reported side effects in the PWS groups. These findings are consistent with a growing body of evidence that PWS supplementation appears to be safe and well-tolerated in apparently healthy individuals [56–59, 69, 70, 72–78] and that combination of these types of nutrients in PWS does not pose undue risk.

## Conclusions

Results suggest that 8-weeks of PWS and PWS + S supplementation can improve some indices of cognitive function and exercise performance during resistance-training without significant side effects in apparently healthy males. However, the inclusion of synephrine did not promote additive benefits. Additional research should investigate the effects of ingesting PWS’s on cognitive and exercise performance, training adaptations, and safety of long-term use in athletes.
